# Comparison of intranasal versus intravenous midazolam for management of status epilepticus in dogs: A multi‐center randomized parallel group clinical study

**DOI:** 10.1111/jvim.15627

**Published:** 2019-10-03

**Authors:** Marios Charalambous, Holger A. Volk, Andrea Tipold, Johannes Erath, Enrice Huenerfauth, Antonella Gallucci, Gualtiero Gandini, Daisuke Hasegawa, Theresa Pancotto, John H. Rossmeisl, Simon Platt, Luisa De Risio, Joan R. Coates, Mihai Musteata, Federica Tirrito, Francesca Cozzi, Laura Porcarelli, Daniele Corlazzoli, Rodolfo Cappello, An Vanhaesebrouck, Bart J.G. Broeckx, Luc Van Ham, Sofie F.M. Bhatti

**Affiliations:** ^1^ Small Animal Department, Faculty of Veterinary Medicine Ghent University Merelbeke Belgium; ^2^ Department of Small Animal Medicine and Surgery University of Veterinary Medicine Hannover Hannover Germany; ^3^ Department of Veterinary Medical Sciences University of Bologna Bologna Italy; ^4^ Department of Clinical Veterinary Medicine Nippon Veterinary and Life Science University Tokyo Japan; ^5^ Department of Small Animal Clinical Sciences Virginia‐Maryland College of Veterinary Medicine Blacksburg Virginia; ^6^ Department of Small Animal Medicine and Surgery, College of Veterinary Medicine University of Georgia Athens Georgia; ^7^ Small Animal Referral Centre Animal Health Trust Newmarket United Kingdom; ^8^ Department of Veterinary Medicine and Surgery, College of Veterinary Medicine University of Missouri Columbia Missouri; ^9^ Department of Clinical Veterinary Medicine, Faculty of Veterinary Medicine University of Agricultural Science and Veterinary Medicine Iasi Iasi Romania; ^10^ Clinica Neurologica Veterinaria NVA Milan Italy; ^11^ Policlinico Veterinario Roma Sud Rome Italy; ^12^ North Downs Specialist Referrals Bletchingley United Kingdom; ^13^ Department of Veterinary Medicine University of Cambridge Cambridge United Kingdom; ^14^ Department of Nutrition, Genetics and Ethology, Faculty of Veterinary Medicine Ghent University Merelbeke Belgium

**Keywords:** benzodiazepines, canine, emergency, epilepsy, nasal

## Abstract

**Background:**

The intranasal (IN) route for rapid drug administration in patients with brain disorders, including status epilepticus, has been investigated. Status epilepticus is an emergency, and the IN route offers a valuable alternative to other routes, especially when these fail.

**Objectives:**

To compare IN versus IV midazolam (MDZ) at the same dosage (0.2 mg/kg) for controlling status epilepticus in dogs.

**Animals:**

Client‐owned dogs (n = 44) with idiopathic epilepsy, structural epilepsy, or epilepsy of unknown origin manifesting as status epilepticus.

**Methods:**

Randomized parallel group clinical trial. Patients were randomly allocated to the IN‐MDZ (n = 21) or IV‐MDZ (n = 23) group. Number of successfully treated cases (defined as seizure cessation within 5 minutes and lasting for ≥10 minutes), seizure cessation time, and adverse effects were recorded. Comparisons were performed using the Fisher's exact and Wilcoxon rank sum tests with statistical significance set at *α* < .05.

**Results:**

IN‐MDZ and IV‐MDZ successfully stopped status epilepticus in 76% and 61% of cases, respectively (*P* = .34). The median seizure cessation time was 33 and 64 seconds for IN‐MDZ and IV‐MDZ, respectively (*P* = .63). When the time to place an IV catheter was taken into account, IN‐MDZ (100 seconds) was superior (*P* = .04) to IV‐MDZ (270 seconds). Sedation and ataxia were seen in 88% and 79% of the dogs treated with IN‐MDZ and IV‐MDZ, respectively.

**Conclusions and Clinical Importance:**

Both routes are quick, safe, and effective for controlling status epilepticus. However, the IN route demonstrated superiority when the time needed to place an IV catheter was taken into account.

**Abbreviations**
BBB: blood‐brain barrier
IN: intranasal
MAD: mucosal atomization device
MDZ: midazolam

## 1 INTRODUCTION

Status epilepticus is an emergency that requires rapid and effective delivery of anti‐seizure drugs. Rapid treatment is crucial to avoid primary and secondary brain injury and systemic complications. Because of the anatomical and physiological properties of the nasal cavity as well as its potential to circumvent the blood‐brain barrier (BBB), the intranasal (IN) route might offer an advantageous and novel way to directly and quickly deliver drugs to the brain to treat various disorders.^1^, ^2^, ^3^, ^4^, ^5^, ^6^, ^7^ Intranasal drug delivery has been widely investigated for anesthetic purposes, and experimental studies have reported that it can be effectively used for analgesia (IN‐fentanyl),^8^ sedation (IN‐xylazine,^9^ IN‐medetomidine/ketamine,^10^ IN‐ketamine,^11^, ^12^) and sedation and opioid reversal (IN‐atipamezole/IN‐naltrexone).^13^

Benzodiazepines (eg, midazolam [MDZ]), are used commonly as a first‐line management option for status epilepticus in humans and dogs.^14^, ^15^, ^16^, ^17^, ^18^, ^19^, ^20^, ^21^, ^22^, ^23^ Midazolam, a water‐soluble benzodiazepine, is considered an effective and safe anti‐seizure drug when administered by the IN, IV, or IM routes.^15^, ^18^, ^20^, ^21^, ^22^, ^23^, ^24^ Intranasal MDZ can be useful or even life‐saving, especially when IV access is not available.^15^, ^25^ Intranasal MDZ has been reported to be an effective and safe choice as a sedative drug in children undergoing diagnostic and minor surgical procedures.^26^, ^27^, ^28^, ^29^ It also has been shown to suppress epileptic spike activity on electroencephalograms of epileptic children.^30^ Based on clinical trials, which evaluated the efficacy of IN‐MDZ, it was concluded that IN‐MDZ was effective and safe for terminating status epilepticus in humans and that it can be used not only by clinicians in the hospital environment but also by families at home.^22^, ^31^

It was recently demonstrated that IN‐MDZ was effective and safe as well as superior to rectally administered diazepam for the management of status epilepticus in dogs with idiopathic epilepsy, structural epilepsy, or epilepsy of unknown origin.^15^ In the present study, we compared MDZ given at the same dose but by different routes (IN or IV) for the treatment of status epilepticus in dogs. Our aim was to provide further evidence of the potential efficacy and safety of IN‐MDZ in dogs with status epilepticus and compare it to the gold standard of IV administration to evaluate if a significant difference existed between the 2 routes in the time needed to terminate the epileptic seizures.

## 2 MATERIALS AND METHODS

The methodology we used was similar to that of a previous trial of IN‐MDZ, which has been described previously.^15^ The current study was an open‐label randomized parallel group clinical trial including client‐owned dogs and approved by the enrolling universities' ethical committees. Owner information and consent forms for the study were completed. Dogs with status epilepticus manifesting generalized or focal epileptic seizures with any type of motor activity (ie, tonic‐clonic or myoclonic) caused by idiopathic epilepsy, structural epilepsy, or epilepsy of unknown origin were included. Dogs with reactive seizures associated with metabolic or toxic causes or dogs that had received any drugs before 5 minutes of continuous epileptic seizure activity had passed were excluded. Classification of epilepsy types, clinical signs, and diagnostic approach were based on the International Veterinary Epilepsy Task Force consensus reports.^32^, ^33^ In particular, for the diagnosis of idiopathic epilepsy, classification into 3 tiers of confidence was performed based on history, signalment, and unremarkable interictal neurological examination, blood tests, brain magnetic resonance imaging, and cerebrospinal fluid analysis.^33^ Status epilepticus was defined as a continuous epileptic seizure lasting more >5 minutes, or ≥2 discrete epileptic seizures between which incomplete recovery of consciousness occurred.^32^ Dogs that manifested status epilepticus were randomly assigned to IN‐MDZ or IV‐MDZ groups, using randomized sealed envelopes. Midazolam was administered at the same dosage for both routes (ie, 0.2 mg/kg) after at least 5 minutes of continuous seizure activity. In the dogs allocated to the IV‐MDZ group, an IV catheter, if not already present, was placed immediately. In the IN‐MDZ group, an IV catheter was placed after MDZ administration to provide IV access. All dogs were treated and remained in a hospital environment for constant observation and monitoring for at least 1 hour after benzodiazepine administration.

## 3 OUTCOME ASSESSMENT

### 3.1 Primary outcomes

The outcome measurements included:

1. “Seizure cessation” time, defined as the time between drug administration and seizure cessation.
2. “Seizure relapse” time, defined as the time between seizure cessation and the next seizure.
3. “Doctor‐to‐drug” time, defined as the time needed by the clinician for preparation and administration of the drug. For the IN‐MDZ group, the “doctor‐to‐drug” time included the time needed for preparation of the mucosal atomization device (MAD) and administration of the MDZ and, for the IV‐MDZ group, the time needed to place an IV catheter (if not placed previously) and for preparation and administration of the MDZ.
4. “Total seizure cessation time” included both the “doctor‐to‐drug” and “seizure cessation time” in order to evaluate if the time needed for the seizures to cease was affected by the preparation and administration of IN‐MDZ or IV‐MDZ and placement of an IV catheter.

Cases were considered successful if “seizure cessation time” was <5 minutes after drug administration and the “seizure relapse time” was >10 minutes.^15^ For the unsuccessful cases, the protocol was no longer applicable and additional anti‐seizure drugs could be given as directed by the clinician in charge.

### 3.2 Secondary outcomes

The outcome measurements included:

1. Complications and adverse effects. Heart rate and rhythm, respiratory rate and pattern, blood pressure (by use of Doppler) and oxygen saturation (by use of pulse oximetry) were measured 5 (T5) and 10 (T10) minutes after drug administration and reported if abnormal. Any other unusual events or adverse effects, such as dyspnea, sneezing, vomiting, as well as sedation or ataxia that occurred within 60 minutes were recorded.
2. Difficulties in administration. Any concerns were recorded by the clinician in charge, with examples including but not limited to difficulties in delivering the MAD into the nostrils or placing an IV catheter in a seizuring dog.
3. Further information, such as history of antiepileptic drugs and duration of dogs' seizure activity before inclusion in the trial, was recorded.

### 3.3 Statistical analysis

As in a previous trial,^15^ the primary outcomes evaluated were the number of successful cases in each group and seizure cessation times. Statistical analysis was conducted using the statistical software R (version 3.5.2). Significance was set at *α* ≤ .05. The number of successfully treated cases per group was compared between the 2 groups (IV or IN) using a Fisher's exact test. The remaining outcomes (“seizure cessation” time, “doctor‐to‐drug” time, and “total seizure cessation” time) were compared using a Wilcoxon rank sum test. Continuous variables are reported as median and range.

## 4 RESULTS

### 4.1 Signalment and baseline characteristics of study subjects and disease characterization

Details of signalment, clinical findings, and disease characteristics of the included cases are provided in Table 1.

**Table 1:** Details of signalment, clinical and disease characteristics in each group

Groups	IN‐MDZ	IV‐MDZ
Breed	Crossbreed (22%), Border Collie (15%), Beagle (9%), GSD (9%), Golden Retriever (9%), Labrador Retriever (4%), Chihuahua (4%), Australian Shepherd Dog (4%), German Shorthaired Pointer (4%), Pincher (4%), Irish Setter (4%), Siberian Husky (4%), Pitbull (4%), Cane Corso (4%)	Crossbreed (32%), CKCS (9%), GSD (9%), Border Collie (9%), Labrador Retriever (9%), Dogo Argentino (4%), Dachshund (4%), Poodle (4%), Shih Tzu (4%), Pekingese (4%), Pug (4%), Siberian Husky (4%), English Bulldog (4%)
Age, median (range), y	6 (0.6‐12)	5 (0.3‐12.6)
Sex	Seven intact and 5 neutered males (57%) and 3 intact and 6 neutered females (43%)	Eight intact and 7 neutered males (65%) and 2 intact and 6 neutered females (35%)
Duration of epileptic seizures prior to trial initiation, median (range), s	480 (310‐3600)	510 (302‐14 400)
Epilepsy etiological classification	Twelve dogs (57%) with idiopathic epilepsy, 6 dogs (29%) with structural epilepsy (neoplasia, 1 dog; MUO, 3 dogs; ischemic encephalopathy, 1 dog; hematoma, 1 dog), and 3 dogs (14%) with epilepsy of unknown origin	Thirteen dogs (56%) with idiopathic epilepsy, 8 dogs (35%) with structural epilepsy (neoplasia, 2 dogs; trauma, 1 dog; MUO, 4 dogs; congenital hydrocephalus, 1 dog), and 2 dogs (9%) with epilepsy of unknown origin
Epileptic seizure type classification	One dog (5%) with focal orofacial epileptic seizures; 20 dogs (95%) with generalized tonic/clonic epileptic seizures	Twenty‐three dogs (100%) with generalized tonic/clonic epileptic seizures
Chronic/maintenance AEDs	Eleven dogs (53%) were not receiving chronic antiepileptic medication; the remaining dogs were receiving phenobarbital monotherapy (5 dogs; 25%), imepitoin monotherapy (2 dogs; 10%), levetiracetam monotherapy (1 dog; 4%), phenobarbital/potassium bromide combination treatment (1 dog; 4%), and phenobarbital/potassium bromide/levetiracetam/clonazepam combination treatment (1 dog; 4%)	Fourteen dogs (62%) were not receiving chronic antiepileptic medication; the remaining dogs were receiving phenobarbital monotherapy (4 dogs; 17%), levetiracetam monotherapy (2 dogs; 9%), phenobarbital/potassium bromide combination therapy (1 dog; 4%), phenobarbital/potassium bromide/levetiracetam/zonisamide combination treatment (1 dog; 4%), and phenobarbital/potassium bromide/levetiracetam/clonazepam combination treatment (1 dog; 4%)
Cluster epilepsy (before occurrence of status epilepticus)	Twelve dogs (60%)	Seven dogs (47%)

Abbreviations: AEDs, antiepileptic drugs; CKCS, Cavalier King Charles Spaniel; GSD, German Shepherd Dog; IN, intranasal; MDZ, midazolam; MUO, meningoencephalitis of unknown origin.

### 4.2 Primary and secondary outcomes

Forty‐nine dogs initially were included but 5 were excluded because they were diagnosed with reactive seizures caused by intoxication. Status epilepticus was terminated within 5 minutes by IN‐MDZ (n = 21) and IV‐MDZ (n = 23) in 76% and 61% of cases, respectively. This difference was not statistically significant (*P* = .34). Seizure cessation time was not significantly different (IN‐MDZ [median, 33 seconds] compared to IV‐MDZ [median, 64.5 seconds]; *P* = .63). However, when the time to place an IV catheter and prepare the medication also was considered (ie, total seizure cessation time), IN‐MDZ (median, 100 seconds) was superior (*P* = .04) to IV‐MDZ (median, 270 seconds). For dogs with idiopathic epilepsy, status epilepticus was terminated in 83% and 69% of the cases by IN‐MDZ and IV‐MDZ, respectively (*P* = .64). The seizure cessation time also was not significantly different (IN‐MDZ [median, 27 seconds] compared to IV‐MDZ [median, 78 seconds]; *P* = .07). However, by adding the time needed to place an IV catheter and prepare the medication (total seizure cessation time), IN‐MDZ (median, 66 seconds) was superior (*P* = .02) to IV‐MDZ (median, 314 seconds). In 21% of the successful IV‐MDZ cases, an IV catheter already had been placed before trial initiation. During treatment, no serious adverse effects, apart from sedation and ataxia, and no important difficulties in preparing and administering the medication in either group were reported. Details about the primary and secondary outcomes for each group are provided in Table 2.

**Table 2:** Summary of the primary and secondary outcomes

	IN‐MDZ				IV‐MDZ				*P* values
Successful cases	Total	IE	SE	EUO	Total	IE	SE	EUO	
	16/21 (76%)	10/12 (83%—out of total number of IE cases; 7/12 dogs, 58% tier I; 5/12 dogs, 42% tier II)	3/6 (50%—MUO, 0/3 dogs, 0%; neoplasia, 1/1 dog, 100%; ischemic 1/1 dog, 100%; hematoma, 1/1 dog, 100%; out of total number of SE cases)	3/3 (100%—out of total number of EUO cases)	14/23 (61%)	9/13 (69%—out of total number of IE cases; 5/13, 38% tier I; 8/13 dogs, 62% tier II)	3/8 (37%—MUO, 0/4 dogs, 0%; hippocampal malformation, 0/1 dog, 0%; trauma, 1/1 dog, 100%; neoplasia, 2/2 dogs, 100%; out of total number of SE cases)	2/2 (100%—out of total number of EUO cases)	
	Total: 95% CI, 50%‐90%				Total: 95% CI, 40%‐80%				.34
	IE: 95% CI, 55%‐95%				IE: 95%CI, 42%‐87%				.64
	SE: 95% CI, 50%–90%				SE: 95%CI, 50%‐90%				NA
	EUO: 95% CI, 44%‐100%				EUO: 95%CI, 34%‐100%				NA
“Seizure cessation” time, median (range), s	All cases: 33 (12‐294)				All cases: 64.5 (8–300)				.63
	IE cases: 27 (12‐280)				IE cases: 78 (10‐300)				.07
“Doctor to drug” time, median (range), s	All cases: 29 (9‐102)				All cases: 186 (24‐822)				.002
	IE cases: 42 (9–102)				IE cases: 196 (29‐822)				.005
“Total seizure cessation” time, median (range), s	All cases: 100.5 (26‐350)				All cases: 270 (32‐1122)				.04
	IE cases: 66 (26‐350)				IE cases: 314 (41‐1122)				.02
“Seizure relapse” time, median (range), s	825 (610‐1962)				766 (628‐1728)				NA
	No relapse (5 dogs; 31%)				No relapse (2 dogs; 14%)				
Adverse effects (within 60 min of drug administration)	Severe sedation and ataxia (14/16 dogs; 88%) dysphoria (2/16 dogs; 12%) and a brief episode of sneezing (1/16 dog; 6%)				Severe sedation and ataxia (11/14 dogs; 79%)				NA
Difficulties in administration (this applies to all the cases, i.e., successful and unsuccessful)	Difficulties in preparing or applying the nasal device (5/21 dogs; 24%)				Difficulties in establishing IV catheter during seizures (9/23 dogs; 39%); in 6/23 dogs (26%), the IV catheter was already placed before the status epilepticus initiation				NA

Abbreviations: CI, confidence interval; EUO, epilepsy of unknown origin; IE, idiopathic epilepsy; IN, intranasal; MDZ, midazolam; NA, not assessed/applicable; SE, structural epilepsy.

## 5 DISCUSSION

Our results indicate that both IN and IV administration of MDZ are effective and safe methods for the management of status epilepticus in dogs. In our study, seizure cessation time for IN‐MDZ compared to IV‐MDZ, at the dosage of 0.2 mg/kg, was not significantly different. However, IN‐MDZ was superior to IV‐MDZ in terminating the epileptic seizures when the time to place an IV catheter was considered. It could be hypothesized that the IN route is a favorable alternative to the IV route, especially in cases in which establishing an IV access is not immediately possible, difficult, or time‐consuming. This could be particularly beneficial for status epilepticus because the prognosis is time‐dependent (ie, prolonged seizures are associated with worse outcome and treatment resistance and require immediate management).^34^ Many clinical and pharmacokinetic studies have shown successful results after IN administration of benzodiazepines, in particular MDZ, in epileptic and normal humans as well as in animals.^15^, ^22^, ^25^, ^31^, ^35^, ^36^, ^37^, ^38^, ^39^, ^40^, ^41^, ^42^, ^43^, ^44^, ^45^, ^46^, ^47^, ^48^, ^49^ Studies in epileptic humans that compared IN to IV administration of benzodiazepines showed that both routes were effective for seizure cessation.^22^, ^31^, ^50^ Specifically, IN‐MDZ was as safe and effective as IV diazepam but, if the time to place an IV catheter was not taken into account, seizures were controlled more quickly with IV diazepam. In our study, we found that IN‐MDZ was quicker than IV‐MDZ, and the difference was significant when the time to place an IV catheter was taken into account. Lastly, a meta‐analysis in human patients concluded that although there was minimal difference in the time interval from drug administration to clinical seizure cessation, which was shorter for diazepam by any route than for non‐IV MDZ by any route, this difference was not clinically relevant.^51^

Increasing interest in IN drug administration as a therapeutic option for brain and systemic diseases derives from the particular anatomical, physiological, and histological characteristics of the nasal cavity. Intranasal administration provides an opportunity for rapid systemic drug absorption and rapid onset of action as well as different and advantageous pathways through which the drug can reach the brain.^3^, ^6^, ^52^, ^53^, ^54^, ^55^, ^56^, ^57^ The canine nasal cavity is divided by the nasal septum into 2 symmetric airways, each including the nasal vestibule, respiratory and olfactory regions.^58^ The nasal vestibule has limited vascularization and permeability, which leads to poor absorption of substances such as drugs.^7^, ^58^, ^59^ In contrast, the respiratory and olfactory regions have high vascularization and good permeability and, therefore, are the main sites of drug absorption.^7^, ^58^ Although lipid‐soluble small molecules can be absorbed more easily from the nasal cavity, many drugs targeting the brain are water‐soluble small molecules or large molecules (>400 Da) that cannot freely pass through various mucosal barriers of the body including the nasal mucosa. Midazolam is water‐soluble (marketed solution pH = 3.5) but, after IN administration, becomes lipid‐soluble (nasal cavity pH = 5.5‐6.5^1^, ^60^), and as a result it can cross the nasal mucosa and pass into the brain with a rapid onset of action.^25^, ^61^, ^62^ After absorption, some amount of the drug will undergo clearance and drainage by the systemic circulation and nasal lymphatic vessels, and might not reach the brain.^52^ The remaining amount passes into the circulation and reaches the BBB without being subject to the first‐pass hepatic metabolism, which can enhance the drug's bioavailability.^22^, ^25^, ^62^, ^63^, ^64^, ^65^, ^66^, ^67^

The BBB is an essential factor limiting the development of new drugs targeting the brain because it can restrict the influx of drugs into the brain. All large molecules (ie, >400 Da) and >98% of small molecules cannot penetrate the BBB^6^, ^54^ and therefore cannot achieve adequate therapeutic concentrations in the brain after IV or PO administration.^68^ Only a few lipid‐soluble small molecules (<400 Da; e.g., benzodiazepines, phenobarbital) can penetrate the BBB by lipid‐mediated free diffusion, treating specific disorders such as epilepsy.^69^ Recent studies, however, showed that some proteins,^56^, ^57^, ^70^ peptides,^71^, ^72^ and oligonucleotides^73^, ^74^ actually could reach the brain after IN administration, which supports the fact that these molecules potentially avoided the BBB. Bypassing the BBB, drugs that might not be able to enter the brain could benefit from IN administration and might require lower doses to be effective with fewer adverse effects.^75^, ^76^ This could be a reason why in our study administration 0.2 mg/kg of MDZ IN resulted in an overall higher number of successfully treated cases compared to IV administration, although there was no statistically significant difference. In addition, bypassing the BBB might be beneficial for dogs with drug‐resistant idiopathic epilepsy, because the BBB plays an important role in developing antiepileptic drug resistance (ie, because of overexpression of drug transporters such as P‐glycoprotein and multidrug‐resistance‐associated protein).^77^, ^78^, ^79^ Some molecules can avoid the BBB, enter the brain, and then be distributed to other brain areas from the point of entry, via the olfactory (within olfactory epithelium) and trigeminal (within respiratory epithelium) nerves.^52^, ^56^, ^57^, ^80^ Various mechanisms of transport via these nerve pathways have been described.^56^, ^57^, ^81^ Final distribution of the drug after brain entry points at the level of the olfactory bulb (via the olfactory nerve and nasal epithelium) and the brainstem (via the trigeminal nerve) to other areas of the brain is likely established by intracellular and extracellular transport mechanisms.^82^, ^83^, ^84^, ^85^

Apart from the properties and advantages that the IN route offers to the administration of drugs that target the brain, another important aspect is the formulation and delivery method of the drug (ie, the nasal device). These factors can influence uptake of the drug by the brain. In our trial, similar to a previous study,^15^ we used the MAD used in humans, to deliver the medication into the nasal cavity. This MAD is a type of a spray device that can be used like a syringe and delivers the drug as a very fine mist of 30‐100 μm particles, enhancing the drug's absorption and bioavailability.^86^, ^87^ In veterinary medicine, because the IN drug delivery route has not yet been well established nor widely investigated, no species‐ or breed‐specific nasal administration devices are available. For epilepsy in dogs, in particular, a device should be designed that can contain an MDZ solution and provide quick and advanced delivery into the entire nasal cavity of the dog. Such a development might further enhance the efficacy of IN‐MDZ in cases of status epilepticus. With regard to the drug, in order to choose the most appropriate formulation, the physiological and chemical properties of the drug as well as the disease that is targeted should be taken into consideration. In our study and the previous^15^ clinical study, the MDZ solution marketed for IN administration was used and showed satisfactory results. Lastly, appropriate training of individuals, and in particular pet owners, on how to correctly prepare and administer the IN drug is crucial for achieving the desirable results.

Our study had some limitations that could have adversely influenced the number of successfully treated cases in both groups. In particular, the underlying cause of the seizures could play a role in the response of the affected dogs to MDZ. Epileptic seizures related to meningoencephalitis of unknown origin, and focal epileptic seizures are negative prognostic factors for status epilepticus in dogs.^88^ Similarly to a previous trial,^15^ all cases of meningoencephalitis of unknown origin and the 1 dog with focal epileptic seizures were unsuccessfully treated, although the small number of dogs in these categories precludes definite conclusions. Lastly, time‐dependent drug‐resistant status epilepticus has been reported.^34^ The anticonvulsant potency of benzodiazepines can decrease by 20‐fold within 30 minutes of continuous seizure activity.^89^ In our study, the dogs' median duration of epileptic seizure activity before the inclusion in the trial was 8 and 8.5 minutes in the IN‐MDZ and IV‐MDZ groups, respectively. This fact might have adversely affected the efficacy of MDZ in both groups compared to a situation in which MDZ had been administered earlier (ie, within 5 minutes), although a larger number of dogs would be necessary to draw firm conclusions. However, the waiting period of at least 5 minutes before administering MDZ was crucial in our study because, otherwise, it could be argued that the epileptic seizures ceased because they might have been self‐limiting (ie, inclusion of non‐status epilepticus cases) and not a consequence of the MDZ administration.

## 6 CONCLUSION

Based on our study, both IN and IV MDZ are effective, quick, and safe first‐line medications for controlling status epilepticus in dogs. Considering that establishing IV access in a dog with status epilepticus might be problematic or delay further treatment, IN‐MDZ could be used as a first‐line option, before IV access, for the treatment of status epilepticus in dogs either at the clinic or by owners at home. Despite some considerations with regard to a drug's absorption and delivery to the brain, the IN route offers several potential advantages. These include rapid use and onset of action, non‐invasive and easy administration, a safe and effective method directly targeting the brain, and the ability to overcome the BBB. Further preclinical and clinical studies (including a larger number of subjects and different devices and drug dosages) focusing on this promising route should be performed to establish this therapeutic route for various brain disorders in dogs.

## CONFLICT OF INTEREST DECLARATION

Andrea Tipold serves as Associate Editor for the Journal of Veterinary Internal Medicine. She was not involved in review of this manuscript.

## OFF‐LABEL ANTIMICROBIAL DECLARATION

Authors declare no off‐label use of antimicrobials.

## INSTITUTIONAL ANIMAL CARE AND USE COMMITTEE (IACUC) OR OTHER APPROVAL DECLARATION

Authors declare no IACUC or other approval was needed.

## HUMAN ETHICS APPROVAL

Authors declare human ethics approval was not needed for this study.
